# Multidrug and Extensively Drug-resistant Tuberculosis in Canada 1997–2008: Demographic and Disease Characteristics

**DOI:** 10.1371/journal.pone.0053466

**Published:** 2013-01-09

**Authors:** Jessica Minion, Victor Gallant, Joyce Wolfe, Frances Jamieson, Richard Long

**Affiliations:** 1 Department of Medical Microbiology & Immunology, University of Alberta, Edmonton, Canada; 2 HIV/AIDS and TB Core Surveillance, Public Health Agency of Canada, Ottawa, Canada; 3 National Reference Centre for Mycobacteriology, National Microbiology Laboratory, Winnipeg, Canada; 4 Public Health Laboratories, Public Health Ontario, (representing the Canadian Public Health Laboratory Networks), Toronto, Canada; 5 Division of Pulmonary Medicine, Department of Medicine, University of Alberta, Edmonton, Canada; Institut de Génétique et Microbiologie, France

## Abstract

**Setting:**

Nationwide Canadian public health surveillance.

**Objective:**

Description of demographic features and disease characteristics of drug-resistant tuberculosis (TB) in Canada over a 12 year period.

**Design:**

Continuous surveillance of all cases of culture-confirmed TB in Canada. Demographic and microbiologic features were analyzed and comparisons between drug-susceptible, multidrug-resistant (MDR), and drug-resistant not-MDR were made. Cases of extensively drug resistant TB are described.

**Results:**

15,993 cases of culture-confirmed TB were reported during the study period. There were 5 cases of XDR-TB, 177 cases of MDR-TB, and 1,234 cases of first-line drug resistance not-MDR. The majority of drug-resistant cases were reported in foreign-born individuals, with drug-resistant cases diagnosed earlier post-arrival in Canada compared to drug-susceptible cases. In MDR-TB isolates, there was a high rate of drug-resistance to other first- and second-line drugs, making reliable empiric therapeutic recommendations for MDR-TB difficult. There was a statistically significant association between both MDR and drug-resistance not-MDR, and the risk of a negative treatment outcome (defined as treatment failure, absconded, or treatment ongoing >3 yrs).

**Conclusion:**

Drug-resistance complicates TB management even in developed nations with well-established TB control programs. The predominantly international origin of drug-resistant cases highlights the need for global strategies to combat TB.

## Introduction

Multidrug-resistant tuberculosis (MDR-TB), defined as TB resistant to both isoniazid (INH) and rifampicin (RIF), complicates TB control efforts by requiring prolonged treatment with drugs that are less potent, more costly and more toxic than standard INH and RIF-based regimens [1], [2]. Extensively drug-resistant tuberculosis (XDR-TB), defined as resistance to INH, RIF, fluoroquinolones and one or more injectable drugs (amikacin, kanamycin, or capreomycin) is yet more difficult to manage. The World Health Organization (WHO) estimates that between 390,000–510,000 cases of MDR-TB occurred worldwide in 2008, accounting for 3.6% of all incident TB cases [3]. In countries performing second-line drug susceptibility testing on MDR-TB isolates, 5.4% of MDR-TB cases were found to be XDR-TB [3].

Over the past 50 years an increasing proportion of the TB cases in Canada are in the foreign-born population [1]. This epidemiologic change is attributable to declining rates of TB in the Canadian-born non-Aboriginal population and a shift in immigration trends from regions of birth with low TB incidence (i.e. Western Europe) to ones with higher incidence (i.e. Asia, Africa). In Canada, each province and territory has local and/or reference laboratory capacity for isolation of *Mycobacterium tuberculosis* complex and performance of first- and second-line drug susceptibility tests (DSTs). Rather than use standardized treatment regimens based on population surveys of local DSTs patterns, TB control programs in Canada use second-line drug susceptibility testing to design individualized treatment regimens, minimizing the amplification of resistance and sparing patients from potentially unnecessary toxic drugs.

In this paper we review the demographic features and disease characteristics of MDR and XDR-TB cases in Canada over the 12 year period from 1997 to 2008.

## Methods

### Databases

TB drug-resistance in Canada is monitored by the Public Health Agency of Canada (PHAC) through two systems: the Canadian Tuberculosis Reporting System and the Canadian Tuberculosis Laboratory Surveillance System. The case and laboratory information used to populate these databases is provided by the respective provincial/territorial programs to PHAC. For the purpose of this analysis, these databases were linked at the case level using four common elements: reporting province/territory, reporting year, date of birth and sex.

### Drug Susceptibility Testing (DST)

During the study period, Canadian laboratories performed routine susceptibility testing of MTB complex isolates using either BACTEC® 460^(^™^)^ or Mycobacteria Growth Indicator Tube^(^™^)^ 960 (Becton Dickinson, Sparks, MD, USA). The National Reference Centre for Mycobacteriology (NRCM, National Microbiology Laboratory, Winnipeg, Manitoba) provided confirmation and reference testing for second-line drugs. All laboratories participated in proficiency testing programs conducted by the NRCM. All testing methods were performed in compliance with recommendations by the Clinical Laboratory Standards Institute (CLSI) [4]. In Canada, all MDR-TB isolates are recommended to undergo first- and second-line DST [1].

### Analysis

For the years 1997–2008 culture-confirmed MDR-TB cases were compared to (i) cases with drug-susceptible TB and (ii) cases with ‘resistant non-MDR-TB’, defined as resistance to one or more first-line anti-tuberculous drugs (in Canada these include INH, RIF, pyrazinamide and ethambutol) but not meeting the definition of MDR-TB. Demographic features for comparison included: age, sex, population group (Canadian-born Aboriginal people including Status and non-Status North American Indians, Métis and Inuit; Canadian-born ‘other’; and foreign-born), disease type (new, retreatment), disease site (respiratory, non-respiratory) and HIV status (positive, negative). Foreign-born cases of MDR-TB were further characterized by WHO TB epidemiological region based on their country of birth [5].

Available DST results for MDR-TB isolates to pyrazinamide, ethambutol and second-line anti-tuberculous agents (including streptomycin, kanamycin, amikacin, capreomycin, ofloxacin, ethionamide, *para*-aminosalicylic acid [PAS], and rifabutin] are reported [6]. It is very likely that more DST was performed than was reported to PHAC; more second-line DST was performed after the initial reports of XDR-TB were published [7], [8].

Clinical outcomes of patients with MDR-TB were compared to (i) outcomes of cases with drug-susceptible TB and (ii) outcomes of cases with ‘resistant non-MDR-TB’. Outcomes were categorized as cure/treatment completed, transferred out (care transferred to another country), death, failure/absconded/treatment ongoing, or other. For foreign-born cases, the time from arrival to diagnosis was calculated by taking the ‘year of diagnosis’ minus ‘year of arrival’.

Information from individual cases of XDR-TB in Canada for the years 1997–2008 was collected from incident jurisdictions and summarized.

### Statistics

Between-group comparisons were made using odds ratios (ORs) and presented with 95% confidence intervals (CIs) calculated using Fisher’s exact methods [9]. Statistical significance is defined as 95% CIs which do not overlap null. Ethics approval was not required as anonymous and routinely collected surveillance data were used.

## Results

There were 15,993 cases of culture-confirmed TB reported in Canada from 1997 to 2008 (Table 1). This represents 78% of all TB cases reported during the study period. Of these, 91.2% (n = 14,582) were drug-susceptible, 7.7% (n = 1,234) were resistant to one or more first-line drugs but did not meet the definition of MDR-TB and 1.1% (n = 177) were MDR-TB. Of the 177 MDR-TB cases five (2.8%) had XDR-TB.

**Table 1:** Demographic features and disease characteristics of culture-positive tuberculosis cases in Canada by drug susceptibility pattern of incident case isolate (1997–2008).

		Drug Susceptibility Pattern			OR (95% CI)†	
Characteristics	Total	Susceptible	Resistant non-MDR*	MDR	MDR vs Susceptible	Resistant non-MDR vs Susceptible
	No. (%)	No. (%)	No. (%)	No. (%)		
**No. Assessed**	15993	14582	1234	177		
**Age (years)**						
0–14	437 (2.7)	416 (2.9)	18 (1.5)	3 (1.7)	1.65 (0.31, 5.68)	0.71 (0.41, 1.16)
15–34	4992 (31.2)	4454 (30.5)	439 (35.6)	99 (55.9)	*5.09 (3.05, 8.95)*	*1.61 (1.37, 1.90)*
35–64	6176 (38.6)	5594 (38.4)	525 (42.5)	57(32.2)	*2.33 (1.35, 4.22)*	*1.53 (1.31, 1.80)*
>64	4388 (27.4)	4118 (28.2)	252(20.4)	18 (10.2)	1.0	1.0
**Sex**						
Male	8784 (54.9)	8035 (55.1)	657 (53.2)	92 (52.0)	1.0	1.0
Female	7209 (45.1)	6547 (44.9)	577 (46.8)	85 (48.0)	1.13 (0.83, 1.54)	1.08 (0.96, 1.21)
**Population Group**						
CBO	2457 (15.4)	2278 (15.6)	170 (13.8)	9 (5.1)	1.0	1.0
CBA	2601 (16.3)	2539 (17.4)	57 (4.6)	5 (2.8)	0.50 (0.13, 1.66)	*0.30 (0.22, 0.41)*
FB	10642 (66.5)	9489 (65.1)	990 (80.2)	163 (92.1)	*4.35 (2.23, 9.69)*	*1.40 (1.18, 1.67)*
Unknown	293 (1.8)	276 (1.9)	17 (1.4)	0 (0)	0 (0, 4.21)	0.83 (0.43, 1.39)
**Disease Type** ‡						
New	14409 (90.1)	13206 (90.6)	1091 (88.4)	112 (63.3)	1.0	1.0
Retreatment	1290 (8.1)	1120 (7.7)	115 (9.3)	55 (31.1)	*5.79 (4.09, 8.11)*	*1.24 (1.01, 1.52)*
Unknown	294 (1.8)	256 (1.8)	28 (2.3)	10 (5.6)	*4.61 (2.12, 8.92)*	1.32 (0.86, 1.97)
**Disease Site**						
Respiratory§	11949 (74.7)	10914 (74.8)	893 (72.4)	142 (80.2)	1.0	1.0
Non-Respiratory^II^	2460 (15.4)	2220 (15.2)	214 (17.3)	26 (14.7)	0.90 (0.57. 1.38)	1.18 (1.00, 1.38)
Unknown	1584 (9.9)	1448 (9.9)	127 (10.3)	9 (5.1)	*0.48 (0.21, 0.94)*	1.07 (0.88, 1.30)
**HIV Status**						
Negative	2844 (17.8)	2628 (18.0)	187 (15.2)	29 (16.4)	1.0	1.0
Positive	508 (3.2)	455 (3.1)	44 (3.6)	9 (5.1)	1.79 (0.74, 3.92)	1.36 (0.94, 1.93)
Unknown	12641 (79.0)	11499 (78.9)	1003 (81.3)	139 (78.5)	1.10 (0.73, 1.70)	*1.23 (1.04, 1.45)*

Abbreviations: No., number; vs, versus; MDR multidrug-resistant; OR odds ratio; CBA Canadian-born Aboriginal; CBO Canadian-born ‘other’; FB foreign-born; HIV human immunodeficiency virus.

* Refers to resistance to one or more first-line drugs but not meeting the definition of MDR.

† Confidence Intervals calculated using Fisher’s exact methods; ORs reaching statistical significance, based on non-overlapping 95% CIs are highlighted in italics.

‡ As defined by the Canadian Tuberculosis Standards (1).

§ includes primary, respiratory, and ‘other respiratory’.

II includes peripheral lymph nodes, miliary, CNS and other non-respiratory.

Compared to cases with drug-susceptible TB, individuals between the ages of 15 and 34 years were the most likely to have drug-resistant TB, followed by those between 35 and 64 years (Table 1). This was true of both MDR-TB and resistant non-MDR-TB. Foreign-born cases had 4.35 (95% CI 2.23–9.69) times higher odds of having MDR-TB compared to Canadian-born non-Aboriginal cases and 1.40 (1.18–1.67) times higher odds of having drug-resistant non-MDR-TB. Further characterisation of the 14 Canadian-born MDR cases is provided in Table S1.

Identification as a retreatment case of TB was a statistically significant risk factor for both MDR and non-MDR drug resistance (OR 5.79 [4.09–8.11] and OR 1.24 [1.01–1.52], respectively; Table 1). Infection with HIV was also associated with an increase in both MDR and non-MDR drug resistance, however this did not reach statistical significance, possibly because of the large proportion of cases with unknown HIV status (79.0%).

**Figure 1:**
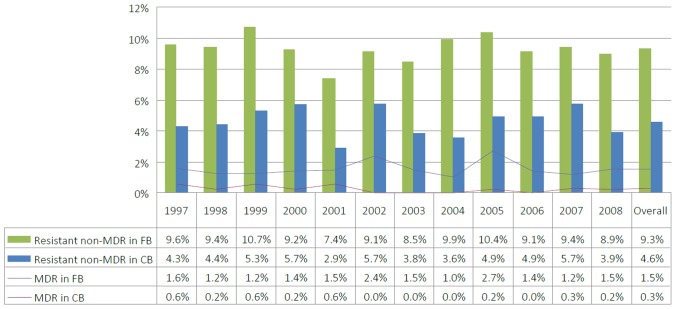
Proportion of culture-positive tuberculosis cases with MDR or non-MDR resistance in Canada (1997–2008). Abbreviations: FB, foreign-born; CB, Canadian-born.

The rates of resistance, both MDR and resistant non-MDR, remained stable during the study period, with rates in the foreign-born population consistently higher than those overall (Figure 1). New TB cases had a lower rate of both MDR and non-MDR resistance (range 0.4–1.2% for MDR, mean = 0.8%; 5.2–9.0% for resistant non-MDR, mean = 7.6%) compared to retreatment cases, which also had more year-to-year variation in resistance rates (range 2.5–10.0% for MDR, mean = 4.3%; 4.4–13.6% for resistant non-MDR, mean = 8.9%).

There were 10,642 cases of foreign-born TB during the study period and region of birth was available for 97.3% (Table 2). The largest proportion of cases occurred in individuals originating from the Western Pacific Region (WPR; 41.8%, n = 4,448), followed by the Southeast Asian Region (SEAR; 19.8%, n = 2,112). Compared to cases born in the Central European Region or Established Market Economies (CEUR/EME), all other WHO regions-of-birth were associated with an increased risk of drug-resistance. The highest risk for MDR-TB was seen in individuals originating from the Eastern European Region (EER) where 2.9% of all TB cases were MDR (OR 4.83, 1.38–18.92), followed by the WPR where 2.0% were MDR (OR 2.90, 1.19–9.20). Individuals originating from one of the 27 high-burden MDR-TB countries accounted for 71.1% of all MDR cases [3]. In order of frequency, the high MDR-TB countries contributing the largest number of MDR-TB cases were the Philippines (32 cases), China (29 cases), Vietnam (18 cases), India (16 cases), and South Korea (8 cases).

**Table 2:** WHO epidemiological region-of-birth of foreign-born culture-positive tuberculosis cases in Canada by drug susceptibility pattern of incident case isolate (1997–2008).

		Drug Susceptibility Pattern			OR (95% CI)‡	
WHO Region	Total No. (%)*	SusceptibleNo. (%)	Resistant non-MDR† No. (%)	MDR No. (%)	(MDR vs S)	(R non-MDR vs S)
**No. Assessed**	10642	9489 (89.2)	990 (9.3)	163 (1.5)		
CEUR/EME	782 (7.3)	734 (93.9)	43 (5.5)	5 (0.6)	1.0	1.0
AFR-High	820 (7.7)	741 (90.4)	65 (7.9)	14 (1.7)	2.77 (0.94, 9.88)	1.50 (0.99, 2.29)
AFR-Low	180 (1.7)	166 (92.2)	12 (6.7)	2 (1.1)	1.77 (0.17, 10.91)	1.23 (0.58, 2.45)
AMR	706 (6.6)	633 (89.7)	66 (9.3)	7 (1.0)	1.62 (0.44, 6.52)	*1.78 (1.17, 2.72)*
EEUR	273 (2.6)	243 (89.0)	22 (8.1)	8 (2.9)	*4.83 (1.38, 18.92)*	1.55 (0.86, 2.70)
EMR	1035 (9.7)	909 (87.8)	110 (10.6)	16 (1.5)	2.58 (0.90, 9.06)	*2.07 (1.42, 3.05)*
SEAR	2112 (19.8)	1944 (92.0)	148 (7.0)	20 (0.9)	1.51 (0.55, 5.17)	1.30 (0.91, 1.89)
WPR	4448 (41.8)	3856 (86.7)	504 (11.3)	88 (2.0)	*2.90 (1.19, 9.20)*	*2.23 (1.61, 3.15)*
Unknown	286 (2.7)	263 (92.0)	20 (7.0)	3 (1.0)	1.67 (0.26, 8.67)	1.30 (0.71, 2.30)

Abbreviations: No., number; vs, versus; CEUR Central European Region; EME Established Market Economies; AFR-high Africa; High HIV; AFR-low Africa Low HIV; AMR Latin America Region; EEUR Eastern European Region; EMR Eastern Mediterranean Region; SEAR Southeast Asian Region; WPR Western Pacific Region.

* Percentages in ‘Total’ column use total number assessed as denominator. Percentages in other columns use row total as denominator.

† Refers to resistance to one or more first-line drugs but not meeting the definition of MDR.

‡ Confidence Intervals calculated using Fisher’s exact methods; ORs reaching statistical significance, based on non-overlapping 95% CIs are highlighted in italics.

**Figure 2:**
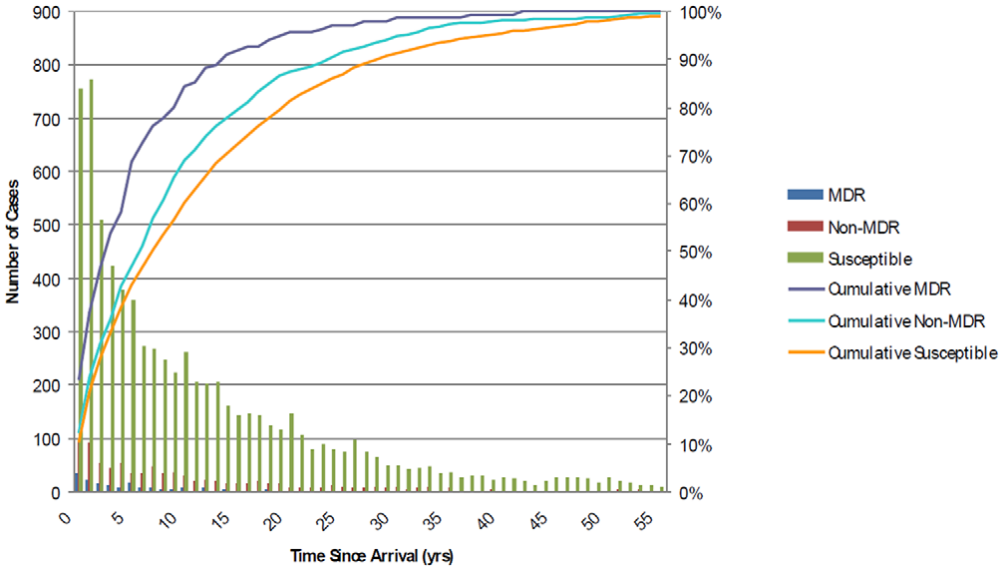
Time from arrival in Canada to diagnosis of foreign-born culture-positive tuberculosis cases by drug susceptiblility pattern of incident case isolate (1997–2008). Time from arrival to diagnosis was calculated by subtracting year of arrival from year of diagnosis. Year of arrival was known for 6928 of the 10589 foreign born cases. Cases with time since arrival between 0–55 years displayed. Bar graph represents the absolute number of cases diagnosed, and line graph represents the cumulative proportion of foreign-born TB cases diagnosed since their time of arrival in Canada.

In foreign-born cases of TB, the time from arrival in Canada until the time of diagnosis was examined by resistance pattern (Figure 2). While the majority of all cases presented within the first 5 years of arrival to Canada, cases with drug-resistance were more likely to present earlier post-arrival than those with drug-susceptible TB. Compared to 38.2% of drug-susceptible cases, 58.2% of MDR cases (p<0.0001) and 42.6% of drug-resistant non-MDR cases (p = 0.017) presented within the first five years. The mean time to diagnosis from arrival for cases of MDR-TB was 5.6 years (median = 3), compared to 9.5 years (median = 6) for drug-resistant non-MDR and 11.7 years (median = 7) for drug-susceptible TB.

Table 3 reports the results of first- and second-line DST for the 177 cases of MDR-TB. Resistance to the remaining first-line agents, pyrazinamide and ethambutol, was seen in 43.4% and 56.1% of MDR isolates, respectively. For those isolates tested, the most common second-line drug resistance was seen with rifabutin (80.5%), streptomycin (68.4%), and ethionamide (38.6%). There were no second-line drugs with less than 5% resistance in MDR isolates, and only kanamycin and amikacin had less than 10% resistance.

**Table 3:** First- and second-line anti-tuberculosis drug-susceptibility test results of MDR tuberculosis case isolates in Canada, 1997–2008.

		Drug Susceptibility Test Results	
Drug	No. Tested (%)	ResistantNo. (%)	SusceptibleNo. (%)
First-Line			
Isoniazid	177 (100)	177 (100)	0 (0)
Rifampin	177 (100)	177 (100)	0 (0)
Pyrazinamide	138 (78.0)	64 (43.4)	74 (53.6)
Ethambutol	148 (83.6)	83 (56.1)	65 (43.9)
**Second-Line** *			
Streptomycin	155 (87.6)	106 (68.4)	49 (31.6)
Kanamycin	59 (33.3)	4 (6.8)	55 (93.2)
Amikacin	97 (54.8)	6 (6.2)	91 (93.8)
Capreomycin	120 (67.8)	13 (10.8)	107 (89.2)
Ofloxacin	116 (65.5)	12 (10.3)	104 (89.7)
Ethionamide	101 (57.1)	39 (38.6)	62 (61.4)
PAS	42(23.7)	5 (11.9)	37 (88.1)
Rifabutin	82 (46.3)	66 (80.5)	16 (19.5)

Abbreviation: No., number; PAS *para*-aminosalicylic acid.

* Some MDR case isolates may have had second-line drug susceptibility testing that was not reported to PHAC.

Of the 177 cases of MDR-TB, 112 (63.3%) were identified as new and 55 (31.1%) were identified as retreatment. There were no statistically significant differences between the proportions of resistant isolates in new cases compared to retreatment cases (Table S2).

Outcome data was available for 57.6% of the cases, including 51.4% of those with MDR-TB (Table 4). In Canada it is uncommon for patients to be labelled as treatment ‘failures’ but rather unresponsive cases are continued on treatment indefinitely or until death or loss to follow-up. For this reason, negative outcomes other than death were combined in a single category: failure, absconded, and treatment ongoing (with a minimum of three years follow-up available). The odds of MDR cases reporting an outcome in this category versus ‘cured’ or ;treatment complete’ was 5.77 (2.87–10.74) times higher than for drug-susceptible cases. The risk for resistant non-MDR cases to report one of these negative outcomes was lower, but still statistically significant (OR 1.65, 1.11–2.39).

**Table 4:** Outcome of treatment of culture-positive tuberculosis cases in Canada by drug susceptibility pattern of incident case isolate (1997–2008).

		Drug Susceptibility Pattern			OR (95% CI)‡	
Outcome*	Total No. (%)	SusceptibleNo. (%)	ResistantNon-MDRNo. (%)†	MDRNo. (%)	(MDR vs S)	(R non-MDR vs S)
**No. Assessed**	15993	14582	1234	177		
Cure/Tx Complete	7582 (47.4)	7006 (48.0)	511 (41.4)	65 (36.7)	1.0	1.0
Transferred Out	262 (1.6)	230 (1.6)	27 (2.2)	5 (2.8)	2.34 (0.73, 5.83)	*1.61 (1.03, 2.43)*
Death	964 (6.0)	904 (6.2)	54 (4.4)	6 (3.4)	0.72 (0.25, 1.65)	0.82 (0.60, 1.10)
Failure/Absconded/Tx Ongoing	290 (1.8)	243 (1.7)	34 (2.8)	13 (7.3)	*5.77 (2.87, 10.74)*	*1.65 (1.11, 2.39)*
Other	117 (0.7)	102 (0.7)	13 (1.1)	2 (1.1)	2.11 (0.25, 8.14)	1.75 (0.89, 3.15)
Unknown	6778 (42.4)	6097 (41.8)	595 (48.2)	86 (48.6)	*1.52 (1.09, 2.14)*	*1.34 (1.18, 1.58)*

Abbreviations: No., number; vs, versus MDR multidrug-resistant; Tx Treatment.

* Outcome definitions: Cure/Tx Complete refers to successful completion of TB therapy, as deemed by their treating physician; Transferred Out refers to patients whose care was initiated in Canada but subsequently continued in another country; Death refers to all-cause mortality during TB treatment; Failure refers to clinical or microbiologic evidence of ongoing disease despite completion of TB treatment; Absconded refers to patients lost to follow-up; Tx ongoing refers to patients remaining on therapy at the time of publication, having had a minimum of 3 years follow-up.

† Refers to resistance to one or more first-line drugs but not meeting the definition of MDR.

‡ Confidence Intervals calculated using Fisher’s exact methods; ORs reaching statistical significance, based on non-overlapping 95% CIs are highlighted in italics.

Of the 177 MDR isolates reported between 1997 and 2008, test results for XDR-TB were reported for 130 (73.4%). Five cases were determined to have XDR-TB (Table 5), with one case experiencing a relapse during this period as previously reported [10]. All cases were in adults between the ages of 39 and 65 years and all presented with pulmonary infection. Three of the five cases were reported in patients who had not previously been treated for TB. Of these five cases, three occurred in foreign-born individuals and one case was acquired abroad by a Canadian-born non-Aboriginal individual. The remaining case occurred in a Canadian-born Aboriginal person and developed locally through acquired secondary drug resistance. Three of the five cases have been classified as cured/treatment complete, and one transferred care to their country of origin before completing therapy. One case of XDR-TB was fatal.

**Table 5:** XDR tuberculosis in Canada (1997–2008): demographic features and case characteristics.

Case	Yearof Dx	Province/Territory	Age(yrs)	Sex	PopulationGroup	Disease Type	Disease Site	HIVStatus	Outcome
1(Episode 1)*	1997	AB	50	F	CBO	New	Pulmonary(smear negative)	Negative	Cured
1(Episode 2)	2001	AB	54	F	CBO	Re-treatment	Pulmonary(smear positive)	Negative	Cured
2	2002	MB	64	F	FB	Re-treatment	Pulmonary(smear positive)	Unknown	Died
3	2003	ON	65	M	FB	Re-treatment	Pulmonary(smear positive)	Unknown	Returned tocountry of origin
4†	2006	ON	39	M	CBA	New	Pulmonary(smear unknown)	Unknown	TreatmentComplete
5	2008	ON	65	F	FB	New	Pulmonary(smear unknown)	Unknown	TreatmentComplete

Abbreviations: Yrs, years; XDR extensively drug-resistant; AB Alberta; MB Manitoba; ON Ontario; F female; M male;

CBO Canadian-born ‘other’; CBA Canadian-born Aboriginal; FB Foreign-born; Dx Diagnosis.

* infection acquired abroad (Spain); her first episode has been reported previously (10).

† infection acquired locally.

## Discussion

Drug resistance has become one of the most challenging aspects of TB control and threatens to become an even greater problem in the future [2]. Globally, 12 countries have reported nation-wide or sub-national proportions of MDR-TB of 6% or higher among new cases of TB with rates as high as 28% in some areas of Eastern Europe [3]. Among previously treated cases of TB, rates of MDR-TB greater than 50% have been reported from five countries in Eastern Europe and Central Asia [3]. Estimates of global XDR-TB rates are hindered by the lack of availability of reliable diagnostics [11], however, data from continuous surveillance and representative surveys estimate 5.4% of MDR-TB cases globally are XDR-TB, with eight countries estimating rates greater than 10% [3].

In this study drug resistance of any kind was uncommon in the Canadian-born. Most reported cases of TB and most drug resistance were in the foreign-born, highlighting how Canada’s TB control efforts are inextricably linked to global TB control and international rates of resistance. Greater than 90% of the MDR-TB cases reported from 1997 to 2008 were in foreign-born individuals and four of the five cases of XDR-TB were acquired abroad. The proportion of TB that is MDR in foreign-born Canadians is lower than what is reported from their respective regions of birth, however, relative rates roughly correspond to those found in the regions of origin (i.e. we report the highest rates of MDR-TB in individuals originating from the EEUR followed by the WPR, with the lowest rates in those born in the CEUR; amalgamated data from these regions similarly shows the highest rates of MDR-TB in EEUR and WPR, with the lowest rates in CEUR). This emphasizes that while increased screening and vigilance in these high-risk populations is warranted, support of international TB control activities is needed to combat this truly borderless epidemic.

In Canada, the rate of treatment completion/cure among MDR-TB cases is higher than that reported globally: 71% of Canadian MDR-TB cases with outcome data available are classified as cured/treatment complete versus 60% globally [3]. This is likely influenced by both the availability of second-line drugs as well as second-line DST, allowing for individualized therapy. It is also possible, given the high proportion of cases with unknown treatment outcome (42%), that the available outcome data are not representative. Future communications, such as the next edition of the Canadian TB Standards, will encourage more complete DST and reporting.

Our DST results on MDR-TB isolates demonstrate how challenging it is to predict drug susceptibility patterns and thus design empiric treatment regimens for MDR-TB [12]–[14]. In the updated WHO *Guidelines for the Programmatic Management of Drug-resistant Tuberc*ulosis, it is recommended that MDR-TB treatment regimens include pyrazinamide, a fluoroquinolone, a parenteral agent (kanamycin, amikacin, capreomycin), ethionamide, and either cycloserine or PAS with the goal of having a minimum of four active agents during the intensive phase of treatment [15]. Although we found that two of the injectable agents (amikacin and kanamycin) had relatively low rates of resistance (6.2% and 6.8% respectively), resistance to fluoroquinolones, widely regarded as the backbone of effective MDR-TB treatment, was seen in greater than one in ten patients (10.3%). Susceptibility to pyrazinamide and ethionamide, the use of which have both been associated with higher rates of cure [15], was also not reliable with rates of resistance of 43.4% and 38.6%, respectively. Given these findings, it is notable that previous treatment with second-line drugs has been found to be a strong and consistent risk factor for resistance to these drugs, including XDR-TB [16].

Complicating the design of MDR-TB treatment regimens is the lack of evidence for many of the currently used critical concentrations used in susceptibility testing [17]. It is established that the BACTEC MGIT 960 and the BACTEC 460 systems provide comparable second-line DST results [18]. The continued development and acceptance of guidelines for testing and reporting of drug susceptibility will ensure the proper assessment of all MDR-TB cases.

Rapid identification of MDR-TB is important for both patient management and infection control purposes. Over 75% of MDR-TB cases globally are reported to be in treatment-naïve patients (67% in Canada) indicating that ongoing transmission is a significant factor contributing to the epidemic [2], [3]. Minimizing the spread of MDR-TB requires not only having a high degree of suspicion to rapidly diagnose TB, but also utilizing diagnostic tools to initiate effective therapy as soon as possible. While new rapid diagnostics have the potential to revolutionize TB control in many high-burden countries, in low-incidence settings such as Canada their use to detect MDR-TB in unselected patients suspected of having TB may not be efficient. However, targeted use of these tests, such as molecular detection of rifampin resistance in isolates from patients at increased risk of MDR-TB due to region-of-origin, previous treatment, MDR-TB contacts, etc., should be strongly considered [19].

We found a statistically significant difference in the time from immigration to Canada to diagnosis of TB in MDR and drug-resistant non-MDR cases compared to drug-susceptible cases. While this could imply an inherent difference in the length of latency between strains with and without drug resistance, more likely this reflects the temporal increase in prevalence of drug-resistant strains latently infecting Canadian immigrants. In other words, individuals infected in their countries of origin ten years ago were more likely to be exposed to drug-susceptible TB compared to individuals infected in their countries of origin more recently. A Canadian population-based study over a more extended period of time supports this conclusion [20]. This should lead practitioners seeing cases of foreign-born TB to be more suspicious of drug-resistance in those immigrating recently compared to those who likely acquired their infection in the more distant past.

This study had certain limitations. First, linkage between the two databases was probabilistic and not based on names or a unique identification number. Nevertheless, the potential for error is judged to be small as questionable links were confirmed by communicating directly with the reporting jurisdiction. Secondly, only culture-positive TB cases were included in this study and thus characteristics associated with culture-negative TB, including HIV infection, non-respiratory TB, and paediatric TB are likely under-represented. Third, the availability of some data, specifically the proportion of cases with unknown treatment outcome (42.4%), was limited. Particularly given that the absence of information on outcome was itself associated with MDR-TB, resultant conclusions should be interpreted with caution.

### Conclusions

The rate of MDR-TB in Canada remained stable during the period 1997–2008 with an overall prevalence of 1.1% among reported culture-positive TB. The predominance of both MDR and drug-resistant non-MDR cases occurred in foreign-born individuals, with the largest number originating from the WPR, reflecting contemporary trends in immigration. While treatment outcomes for MDR-TB in Canada compare favourably to those reported internationally, 20.9% of cases with known outcomes experienced treatment failure, absconded from treatment, remained on therapy at least three years post-diagnosis, or died. This highlights the seriousness of disease due to drug-resistant TB and emphasizes the influence of the global TB epidemic on Canadian TB control.

## Supporting Information

Table S1 **Profile of Canadian-born MDR cases (n = 14).**
(DOCX)

Table S2 **Anti-tuberculous drug-susceptibility test results of MDR tuberculosis case isolates, by disease type (new vs. retreatment).**
(DOCX)
