# Supplementary material for: Multidrug and Extensively Drug-resistant Tuberculosis in Canada 1997–2008: Demographic and Disease Characteristics
Source: PLoS One. 2013 Jan 9;8(1):e53466. doi: 10.1371/journal.pone.0053466 (PMC3541271; doi:10.1371/journal.pone.0053466)
Supplement: Table S1 — Profile of Canadian-born MDR cases (n = 14). (DOCX) [file pone.0053466.s001.docx]

**Table S1. Profile of Canadian-born MDR cases (n=14)**

|  |  | **Non-Aboriginal (n=9)** | **Aboriginal (n=5)** |
| --- | --- | --- | --- |
| **Sex** | Male | 4 | 5 |
|  | Female | 5 | - |
| **Age** | Median | 37 | 43 |
|  | Range | 2-87 | 33-71 |
| **HIV status** | Positive | 1 | - |
|  | Negative | 4 | 5 |
|  | Unknown | 4 | - |
| **First Episode** | Yes | 7 | 3 |
|  | No | 1* | 1† |
|  | Unknown | 1 | 1 |
| **Diagnosis** | Pulmonary | 6 | 4 |
|  | Other Respiratory | 1‡ | - |
|  | Peripheral Lymph Node | 1 | - |
|  | Miliary | 1 | - |
|  | Other | - | 1§ |
| **Outcome** | Treatment Complete | 7 | 5 |
|  | Death | 1^¶^ | - |
|  | Other | 1 | - |

*Previous episode 1997

†Previous episode 1976

‡Tuberculosis of intrathoracic lymph nodes

§Tuberculosis of bone/joint

^¶^TB did not contribute to death
