# Supplementary material for: Multidrug and Extensively Drug-resistant Tuberculosis in Canada 1997–2008: Demographic and Disease Characteristics
Source: PLoS One. 2013 Jan 9;8(1):e53466. doi: 10.1371/journal.pone.0053466 (PMC3541271; doi:10.1371/journal.pone.0053466)
Supplement: Table S2 — Anti-tuberculous drug-susceptibility test results of MDR tuberculosis case isolates, by disease type (new vs. retreatment). (DOCX) [file pone.0053466.s002.docx]

**Table S2. Anti-tuberculous drug-susceptibility test results of MDR tuberculosis case isolates, by disease type (new vs. retreatment)**

|  | **New Cases**  **(n=112)** | | | **Re-treatment Cases**  **(n=55)** | | | **Unknown**  **(n=10)** | | |
| --- | --- | --- | --- | --- | --- | --- | --- | --- | --- |
| **Drug** | **R (%)** | **S (%)** | **NA (%)** | **R**  **(%)** | **S**  **(%)** | **NA**  **(%)** | **R**  **(%)** | **S**  **(%)** | **NA**  **(%)** |
| **First-line** | | | | | | | | | |
| Isoniazid | 112(100) | 0(0) | 0(0) | 55(100) | 0(0) | 0(0) | 10(100) | 0(0) | 0(0) |
| Rifampicin | 112(100) | 0(0) | 0(0) | 55(100) | 0(0) | 0(0) | 10(100) | 0(0) | 0(0) |
| Pyrazinamide | 41(36.6) | 48(42.9) | 23 (20.5) | 19(34.5) | 24(43.6) | 12(21.8) | 4(40.0) | 2(20.0) | 4(20.0) |
| Ethambutol | 49(43.8) | 43(38.4) | 20 (17.9) | 28(50.9) | 20(36.4) | 7(12.7) | 6(60.0) | 2(20.0) | 2(20.0) |
| **Second-line** | | | | | | | | | |
| Streptomycin | 68(60.7) | 30(26.8) | 14 (12.5) | 33(60.0) | 16(29.1) | 6(10.9) | 5(50.0) | 3(30.0) | 2(20.0) |
| Kanamycin | 2(1.8) | 34(30.4) | 76 (67.9) | 2(3.6) | 18(32.7) | 35(63.6) | 0(0.0) | 3(30.0) | 7(70.0) |
| Amikacin | 3(2.7) | 57(50.9) | 52 (46.4) | 2(3.6) | 28(50.9) | 25(45.5) | 1(10.0) | 6(60.0) | 3(30.0) |
| Capreomycin | 7(6.3) | 68(60.7) | 37 (33.0) | 5(9.1) | 33(60.0) | 17(30.9) | 1(10.0) | 6(60.0) | 3(30.0) |
| Ofloxacin | 5(4.5) | 68(60.7) | 39(34.8) | 6(10.9) | 30(54.5) | 19(34.5) | 1(10.0) | 6(60.0) | 3(30.0) |
| Ethionamide | 26(23.2) | 40(35.7) | 46(41.1) | 10(18.2) | 22(40.0) | 23(41.8) | 3(30.0) | 0(0.0) | 7(70.00 |
| PAS | 4(3.6) | 24(21.4) | 84(75.0) | 1(23.6) | 13(1.8) | 41(74.5) | 0(0.0) | 0(0.0) | 10(100.0) |
| Rifabutin | 38(33.9) | 12(10.7) | 62(55.4) | 25(45.5) | 4(7.3) | 26(47.3) | 3(30.0) | 0(0.0) | 7(70.0) |

R resistant, S susceptible, NA not available/not tested
